# The central regulator HIF-1α in digestive system pathologies: inflammation and cancer

**DOI:** 10.3389/fimmu.2026.1772110

**Published:** 2026-03-31

**Authors:** Guoyou Gou, Min Wen, Guonian Li, Feihong Shu, Fang Wang, Youjia Liu, Rui Xie, Jingyu Xu

**Affiliations:** 1 Zunyi Medical University, Zunyi, China; 2 Department of Endoscopy and Digestive System, Guizhou Provincial People’s Hospital, Guiyang, Guizhou, China; 3 Collaborative Innovation Center for Tissue Repair and Regenerative Medicine (MOE), Zunyi Medical University, Zunyi, Guizhou, China

**Keywords:** cancer, digestive, HIF-1α, inflammation, tumor-associated immune cells

## Abstract

Hypoxia-inducible factor-1α (HIF-1α) is a critical transcriptional regulator that allows cells to detect and respond to hypoxic conditions. Its stabilized expression and prolonged activation significantly contribute to the development and progression of inflammatory diseases and malignancies within the digestive system. In the context of inflammation, HIF-1α plays a pivotal role in the pathogenesis of various disorders—including periodontitis, eosinophilic esophagitis, viral hepatitis, pancreatitis, and inflammatory bowel disease—by modulating the expression of inflammatory mediators, driving cellular metabolic reprogramming, and influencing tissue barrier function. In terms of tumor biology, this review synthesizes pan-cancer analysis data to summarize the expression patterns of HIF-1α in major gastrointestinal malignancies, including esophageal, gastric, pancreatic, hepatocellular, and colorectal cancers. It elucidates HIF-1α’s role in directly promoting tumor cell proliferation, invasion, and drug resistance, while also remodeling the tumor microenvironment to regulate immune responses. We dissect how HIF-1α cooperatively drives the formation of an immunosuppressive microenvironment, thereby influencing the efficacy of immunotherapy. This review aims to integrate the regulatory networks and molecular mechanisms of HIF-1α in digestive system inflammation, tumors, and their immune microenvironment, ultimately providing novel theoretical foundations and strategic insights for targeted therapies and immune interventions in related diseases.

## 1 Introduction

Hypoxia-inducible factor-1α (HIF-1α) is a master transcriptional regulator that enables cellular adaptation to hypoxic conditions through oxygen-sensitive hydroxylation. Under normoxia, prolyl hydroxylase domain (PHD) enzymes hydroxylate HIF-1α, leading to its recognition by the von Hippel–Lindau (VHL) E3 ubiquitin ligase and subsequent proteasomal degradation. In contrast, hypoxia suppresses PHD activity, allowing HIF-1α to stabilize, translocate to the nucleus, and dimerize with HIF-1β. The resulting heterodimer binds to hypoxia-response elements (HREs) and activates a broad transcriptional program that promotes angiogenesis, metabolic reprogramming toward glycolysis, cell survival, and immune modulation (1). These hypoxia-adaptive responses are deeply implicated in digestive pathophysiology, positioning HIF-1α as a key molecular node at the intersection of inflammation and tumorigenesis (2–5).

Under physiological conditions, HIF-1α is essential for maintaining the integrity of the digestive mucosal barrier and immune homeostasis. For instance, in ulcerative colitis, the activation of HIF-1α in epithelial cells has been shown to exert protective and reparative effects (6, 7). However, under sustained or aberrant pathological stimulation, such as metabolic stress, stromal crosstalk, and therapeutic pressure, the stabilization and activation of HIF-1α emerge as crucial drivers of malignant transformation. This process is predominantly mediated by a self-reinforcing positive feedback loop that connects cancer progression, hypoxia, and HIF-1α signaling. This loop further cultivates a pro-tumorigenic microenvironment that enhances tumor adaptation, particularly through the Warburg effect (8, 9).

In digestive system tumors, the oncogenic effects of HIF-1α are multifaceted. On one hand, it drives metabolic reprogramming by inducing the Warburg effect and upregulating glucose transporters and glycolytic enzymes. This allows tumor cells to preferentially rely on glycolysis for energy, even under aerobic conditions, thereby supporting their rapid proliferative needs (10, 11). On the other hand, HIF-1α serves as a crucial driver of angiogenesis and invasion-metastasis. It promotes tumor neovascularization by upregulating vascular endothelial growth factor (VEGF) and enhances cellular migratory capacity through the induction of epithelial-mesenchymal transition and related protein expression. These mechanisms are closely associated with the progression of gastric and esophageal cancers (12–14). HIF-1α plays a significant role in tumor-associated immune cells by promoting the recruitment and polarization of myeloid-derived suppressor cells and tumor-associated macrophages, thereby facilitating tumor immune evasion (15, 16). In addition to the aforementioned mechanisms, HIF-1α contributes to therapy resistance by attenuating DNA damage, enhancing DNA repair capacity, and boosting the antioxidant defenses of tumor cells, collectively increasing their resistance to radiotherapy and chemotherapy (17).

Given its critical role, this review aims to synthesize the functions of HIF-1α in digestive system inflammation, tumor progression, and tumor-associated immune cells. It provides a comprehensive analysis of its mechanisms within the inflammatory and tumor immune microenvironment, with the goal of evaluating its potential as a biomarker for guiding personalized combination therapies and as a therapeutic target. Ultimately, this work seeks to offer new scientific insights for clinical treatment strategies.

## 2 HIF-1α and digestive system inflammatory diseases

### 2.1 Periodontitis and pulpitis

Periodontitis is a chronic inflammatory disease influenced by multiple factors, among which hypoxia, excessive oxidative stress, and immune dysregulation are the most prominent (18). Patients with periodontitis show elevated levels of HIF-1α, HIF-2α, HIF-3α, and VEGF in gingival crevicular fluid compared to healthy controls (19, 20). Consequently, investigating HIF-1α and VEGF presents a promising avenue for enhancing the management of periodontitis. Recent studies have shown that periodontal ligament stem cells isolated from patients with periodontitis, when stimulated with the pro-inflammatory cytokines TNF-α and IL-1β, exhibit stabilization of HIF-1α and activation of ubiquitin C-terminal hydrolase L1 (UCHL1). This process results in increased expression and nuclear translocation of Yes-associated protein (YAP, a transcriptional co-activator involved in organ size control and regeneration), which ultimately promotes the secretion of elevated levels of VEGFA and angiopoietin-1 by these stem cells. Conversely, the inhibition of UCHL1 has been demonstrated to reduce the infiltration of CD3^+^ T cells and CD20^+^ B cells, decrease the levels of IL-6 and IFN-γ, and mitigate alveolar bone resorption (21, 22). Furthermore, within the hypoxic microenvironment induced by periodontitis, the hypoxia-associated long non-coding RNA 01126 suppresses the proliferation of periodontal ligament cells while promoting their apoptosis and inflammatory responses via the miR-518a-5p/HIF-1α/MAPK signaling axis, thereby contributing to the progression of periodontitis. Mechanistic studies have shown that LINC01126 is mainly localized in the cytoplasm and acts as a molecular sponge to adsorb miR-518a-5p, thereby relieving the post-transcriptional inhibition of its target gene HIF-1α by miR-518a-5p. The accumulation of HIF-1α can further activate the downstream mitogen-activated protein kinase(MAPK) signaling pathway (including p38, JNK, and ERK), ultimately leading to decreased proliferation, increased apoptosis, and elevated secretion of inflammatory cytokines (such as IL-1β and TNF-α) in periodontal ligament cells (23).

In addition to periodontitis, pulpitis represents another common oral inflammatory disease, primarily caused by bacterial infection. Research has notably focused on dental pulp stem cells and dental pulp fibroblasts. Under hypoxic conditions induced by cobalt chloride, miR-143-5p is downregulated in dental pulp stem cells, leading to the upregulation of HIF-1α and retinoic acid receptor-related orphan receptor alpha (RORA), which ultimately enhances the pro-angiogenic capacity of these cells (24). Regarding dental pulp fibroblasts, miR-22-3p inhibits the secretion of pro-inflammatory cytokines mediated by the NOD-, LRR- and pyrin domain-containing protein 3 (NLRP3)/caspase-1 (CASP1) inflammasome pathway through targeting both NLRP3 and HIF-1α (25). Another study indicates that the NLRP3 inflammasome is not activated by stimulation with either Porphyromonas gingivalis LPS or hypoxia alone; however, under the combined stimulation of hypoxia and P. gingivalis LPS, inflammasome activation is markedly enhanced, thereby promoting the expression levels of IL-1β and HIF-1α (26).

### 2.2 Eosinophilic esophagitis

In eosinophilic esophagitis, localized hypoxia in the esophageal mucosal epithelium leads to dysregulation of HIF-1α. This dysregulation contributes to impaired barrier function through two primary mechanisms. First, it induces transcriptional suppression of genes such as CLDN1, GLUT1, PGK1, ADM, VEGFA, and ENO1, resulting in reduced tight junctions between epithelial cells. Second, prolonged hypoxia leads to an adaptive decline in the expression of the transcription factor HIF-1α. As a key downstream target of HIF-1α, CD73 (encoded by NT5E) is subsequently downregulated. CD73 serves as the rate-limiting enzyme for extracellular adenosine production, and its reduced expression directly diminishes adenosine generation in the extracellular milieu, thereby impairing adenosine-mediated signaling. This attenuation of signal transduction—particularly through its primary receptor adenosine A2B receptor (ADORA2B)—compromises the activation of protective epithelial responses. Specifically, suppression of the intracellular cyclic AMP (cAMP)/cAMP response element-binding protein (CREB) signaling pathway reduces the expression of tight junction proteins such as Occludin, disrupting epithelial barrier integrity. Concurrently, impaired fibronectin responses hinder re-epithelialization during wound repair, this directly disrupts both the physical barrier and repair capacity of the esophageal epithelium, ultimately leading to severe impairment of barrier integrity (27, 28).

Beyond barrier dysfunction, HIF-1α dysregulation also leads to defective differentiation of esophageal epithelial cells. In HIF-1α-knockdown EPC2-hTERT cells, increased expression of mitochondrial metabolism/oxidative phosphorylation (OXPHOS) genes and decreased glycolytic capacity were observed. These cells exhibited a significant loss of terminal epithelial differentiation markers, such as involucrin, alongside suppressed glucose transporter expression. Notably, the HIF-1α-deficient phenotype could be rescued by the pan-prolyl hydroxylase inhibitor dimethyloxalylglycine, which restored the expression of epithelial differentiation markers (29).

### 2.3 Hepatitis B virus infection

Chronic hepatitis B virus (HBV) infection induces persistent liver inflammation, which subsequently disrupts hepatic oxygenation and promotes immune activation—a process involving HIF-1α. In this context, the stabilization of HIF-1α reduces RelB protein levels. Clinical sample analysis revealed that HIF1α expression positively correlated with HBcAg-positive regions, whereas A3B mRNA levels were significantly reduced in these areas, suggesting that the HIF1α-established microenvironment may enable HBV to evade immune clearance (29, 30). Mechanistic studies demonstrated that HIF1α stabilization markedly reduces RelB protein levels through post-transcriptional regulation—as evidenced by unchanged RelB mRNA accompanied by decreased protein expression—thereby impairing NF-κB-mediated A3B transcriptional activation. Proteomic data indicated that pathways involved in RNA processing and ribosomal function are compromised under hypoxic conditions, implying potential blockade of RelB mRNA nuclear export or translation; additionally, RelB protein may undergo accelerated degradation via the proteasome pathway (31). Of note, HIF-1α-mediated suppression of RelB occurs independently of its canonical partner ARNT, suggesting a direct protein-protein interaction between HIF-1α and RelB. Elucidation of this mechanism provides new insights for optimizing CHB immunotherapy: combination with HIF1α inhibitors may relieve the suppression of the RelB/A3B axis, thereby restoring immune-mediated cccDNA clearance (32–34).

### 2.4 Pancreatitis

In chronic pancreatitis, pancreatic fibrosis is a hallmark pathological feature. As fibrosis progresses, the formation of connective tissue and remodeling of blood vessels create a hypoxic microenvironment within the pancreas. This hypoxia induces the expression of HIF-1α, which upregulates the transcription of SPHK1 in pancreatic acinar cells. As a key mediator, the sphingosine kinase 1 (SPHK1)/sphingosine-1-phosphate (S1P) signaling axis derived from pancreatic acinar cells (PACs) has emerged as a central driver of this crosstalk. In response to injurious stimuli such as cerulein or pancreatic duct ligation, PACs upregulate SPHK1 expression, leading to the generation and extracellular release of S1P. Acting in a paracrine manner, S1P engages its cognate receptor S1PR2—abundantly expressed on PSCs—to trigger the AMPK-mTOR signaling cascade, thereby inducing autophagic flux in PSCs. This autophagic switch is a critical event in the transdifferentiation of PSCs into a matrix-producing myofibroblast-like phenotype, ultimately driving extracellular matrix deposition and fibrotic progression. Notably, the resultant tissue hypoxia further amplifies this pathway by stabilizing hypoxia-inducible factors (HIF-1α and HIF-2α) within PACs, which transcriptionally enhance SPHK1 expression. This establishes a self-perpetuating positive feedback loop—termed the “acinar injury–PSC activation–hypoxia–acinar re-activation” cycle—that sustains and amplifies fibrogenesis in chronic pancreatitis (35).

In autoimmune pancreatitis, HIF1A+ classical monocyte-derived macrophages exhibit elevated expression of HIF-1α protein. HIF-1α binds to the promoter region of the NAMPT gene, thereby upregulating its transcription and leading to increased synthesis and secretion of visfatin (NAMPT). As an endogenous ligand, this molecule binds to the TLR4 receptor on the surface of PSCs, triggering an NADPH oxidase-dependent burst of reactive oxygen species (ROS). Acting as a second messenger, ROS facilitate the release of active TGF-β—either by oxidizing the latency-associated peptide (LAP) complex of latent TGF-β or through protease activation—thereby initiating the TGF-β/Smad3 signaling pathway. This cascade drives the transdifferentiation of PSCs into α-SMA^+^ myofibroblasts and promotes excessive extracellular matrix deposition (36).

### 2.5 Inflammatory bowel disease

The intestinal microenvironment is characterized by a steep oxygen gradient, maintained in part by the microbiota, which creates a physiologically hypoxic state essential for homeostasis. This physiological hypoxia supports barrier function largely through the HIF pathway (37).

Under pathological conditions such as Crohn’s disease and spinal cord injury, HIF-1α can be activated by various signals, including hypoxia and pathogen infection (38). Once activated, HIF-1α exerts its functions through two primary pathways. First, as a transcription factor, it collaborates with signaling molecules, such as NF-κB, to upregulate target genes like VEGF, which directly drives angiogenesis and inflammatory responses. Second, it enhances the expression of ABC efflux transporters (including MDR1 and MRP4), thereby altering the intracellular metabolic milieu. This alteration impairs the ability of Th17 cells to effectively respond to immunomodulatory signals from the aryl hydrocarbon receptor (AhR), indirectly weakening immunoregulatory function and perpetuating the inflammatory state (39, 40).

In ulcerative colitis (UC), HIF-1α plays a complex, cell-type-specific central role, akin to a “double-edged sword.” Within intestinal epithelial cells, HIF-1α primarily exerts a protective effect. This protective role is part of a broader program where HIF-1α strengthens barrier function by regulating tight junction proteins, mucins, and antimicrobial peptides, and by supporting energy metabolism during wound repair (37). Studies have confirmed significant ferroptosis features in the intestinal epithelium of UC patients and DSS-induced model mice, characterized by the accumulation of lipid peroxidation products, iron overload, and downregulation of key inhibitory proteins such as GPX4. HIF-1α directly binds to the promoter region of GPX4, positively regulating its transcription, thereby enhancing cellular capacity to clear lipid peroxidation, inhibiting epithelial ferroptosis, and maintaining intestinal barrier integrity (7, 41).

UC involves immune dysregulation, in which the aberrant differentiation of CD4^+^T cells toward a pro-inflammatory Th17 phenotype plays a pivotal role. The mTOR signaling pathway serves as a central hub in this process. Within the inflammatory microenvironment of the gut, activated CD4^+^T cells engage the PI3K-Akt pathway to activate mTORC1. Once activated, mTORC1 specifically enhances the translation and stability of hypoxia-inducible factor 1α (HIF-1α). Acting as a crucial link between metabolism and immunity, HIF-1α translocates to the nucleus to upregulate the expression of the glucose transporter Glut1, thereby reprogramming T cell metabolism toward aerobic glycolysis. Concurrently, HIF-1α directly activates the Th17-lineage master transcription factor RORγt and facilitates the degradation of Foxp3, the key transcription factor for regulatory T cells (Tregs), via the ubiquitin-proteasome pathway. This disrupts the Th17/Treg balance. Consequently, this molecular axis drives the secretion of large amounts of IL-17 and other inflammatory cytokines by Th17 cells, which recruit neutrophils and compromise the intestinal epithelial barrier, ultimately exacerbating colonic mucosal inflammation (42).

The microbiota adds another layer of regulation. Adherent-invasive Escherichia coli (AIEC) produce the metallophore yersiniabactin (Ybt), which sequesters zinc from macrophages. This zinc depletion inhibits prolyl hydroxylase enzymes, stabilizing HIF-1α and driving expression of profibrotic genes like Mmp9. The presence of these HIF-1α^+^ macrophages in fibrotic lesions from Crohn’s disease patients directly links microbiota-mediated HIF-1α activation to intestinal fibrosis (43).

Furthermore, the therapeutic efficacy of cyclosporine A (CsA) in acute severe ulcerative colitis (ASUC) is primarily attributed to its ability to metabolically reprogram neutrophils, shifting them from a pro-inflammatory, tissue-destructive state to a quiescent, surveillance mode. This transition preserves their antimicrobial capacity while minimizing collateral tissue damage. At the molecular level, CsA downregulates sirtuin 6 (SIRT6) expression in neutrophils, thereby relieving SIRT6-mediated inhibition of the transcription factor HIF-1α. This leads to the activation of HIF-1α and its downstream glycolytic targets, fructose-2, 6-biphosphatase 3 (PFKFB3) and pyruvate dehydrogenase kinase 4 (PDK4). This signaling axis drives a metabolic shift toward aerobic glycolysis in neutrophils, providing the energy support necessary for cell survival. Concurrently, CsA suppresses the expression of the chemokine CCL3, reducing neutrophil migratory capacity, and attenuates the release of inflammatory mediators, including ROS, myeloperoxidase (MPO), antimicrobial peptides, and IL-8. Additionally, CsA upregulates caspase recruitment domain-containing protein 8 (CARD8), a negative regulator of inflammation. This functional remodeling—characterized by enhanced metabolism yet subdued effector functions—enables neutrophils to remain viable but quiescent within inflammatory foci, thereby averting epithelial barrier damage caused by excessive activation while preserving fundamental immune surveillance capabilities. Ultimately, through the SIRT6–HIF-1α–glycolysis axis, CsA restores neutrophil homeostasis, alleviates mucosal injury, and induces clinical remission in ASUC (44).

**Figure 1:**
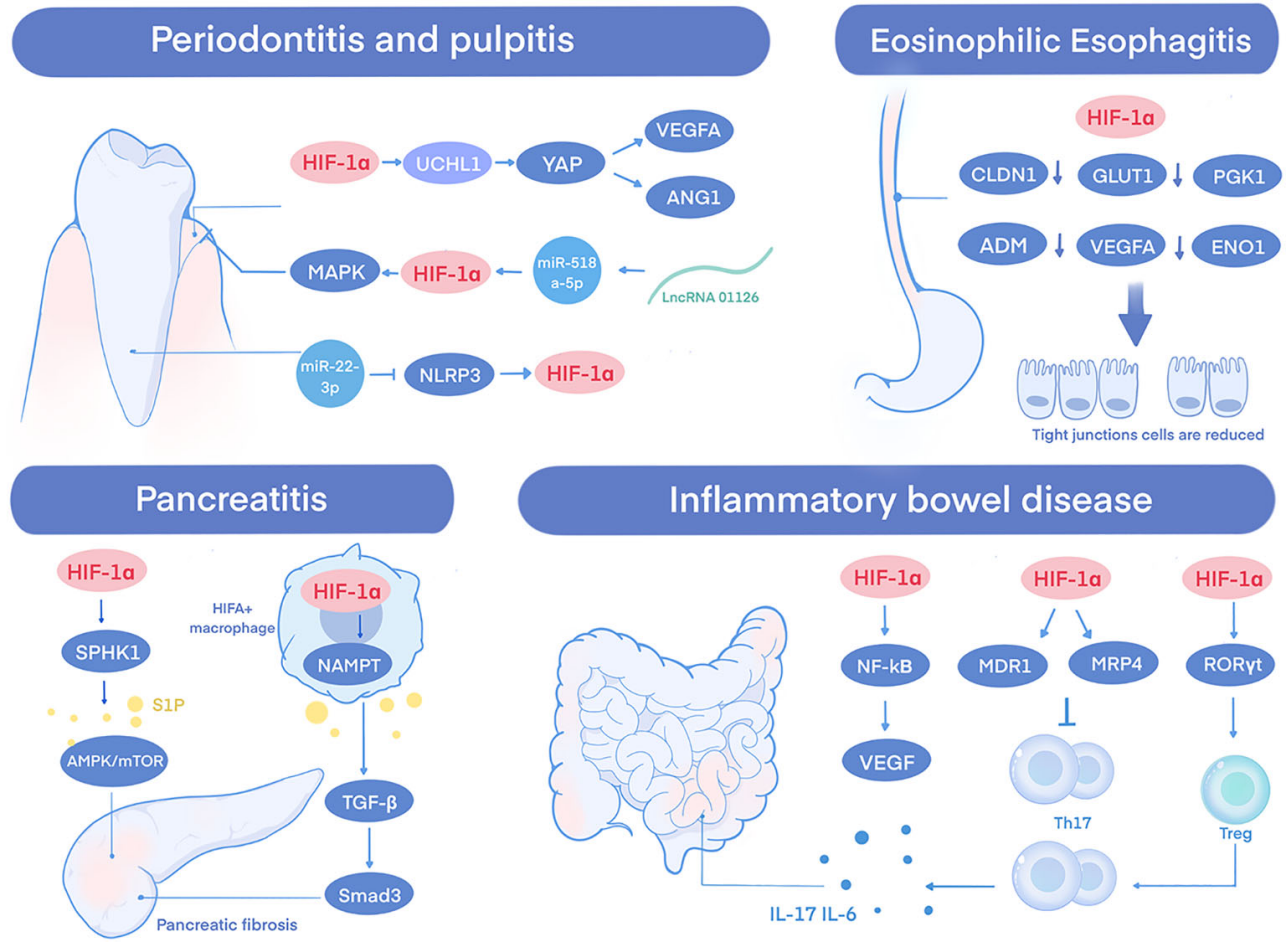
Mechanisms of HIF-1α in digestive system inflammatory diseases. This schematic illustrates how hypoxia-induced HIF-1α activation orchestrates divergent outcomes in inflammatory conditions. The figure highlights key cell types and molecular pathways through which HIF-1α integrates hypoxic stress with tissue inflammation and repair.

This section analyzes the pathophysiological role of HIF-1α across multiple digestive inflammatory conditions, such as periodontitis, pulpitis, eosinophilic esophagitis, viral hepatitis, pancreatitis, and inflammatory bowel disease. It thereby connects hypoxia-driven signaling to disease advancement and highlights novel therapeutic opportunities (**Figure 1**).

## 3 HIF-1α and digestive system tumors

**Figure 2:**
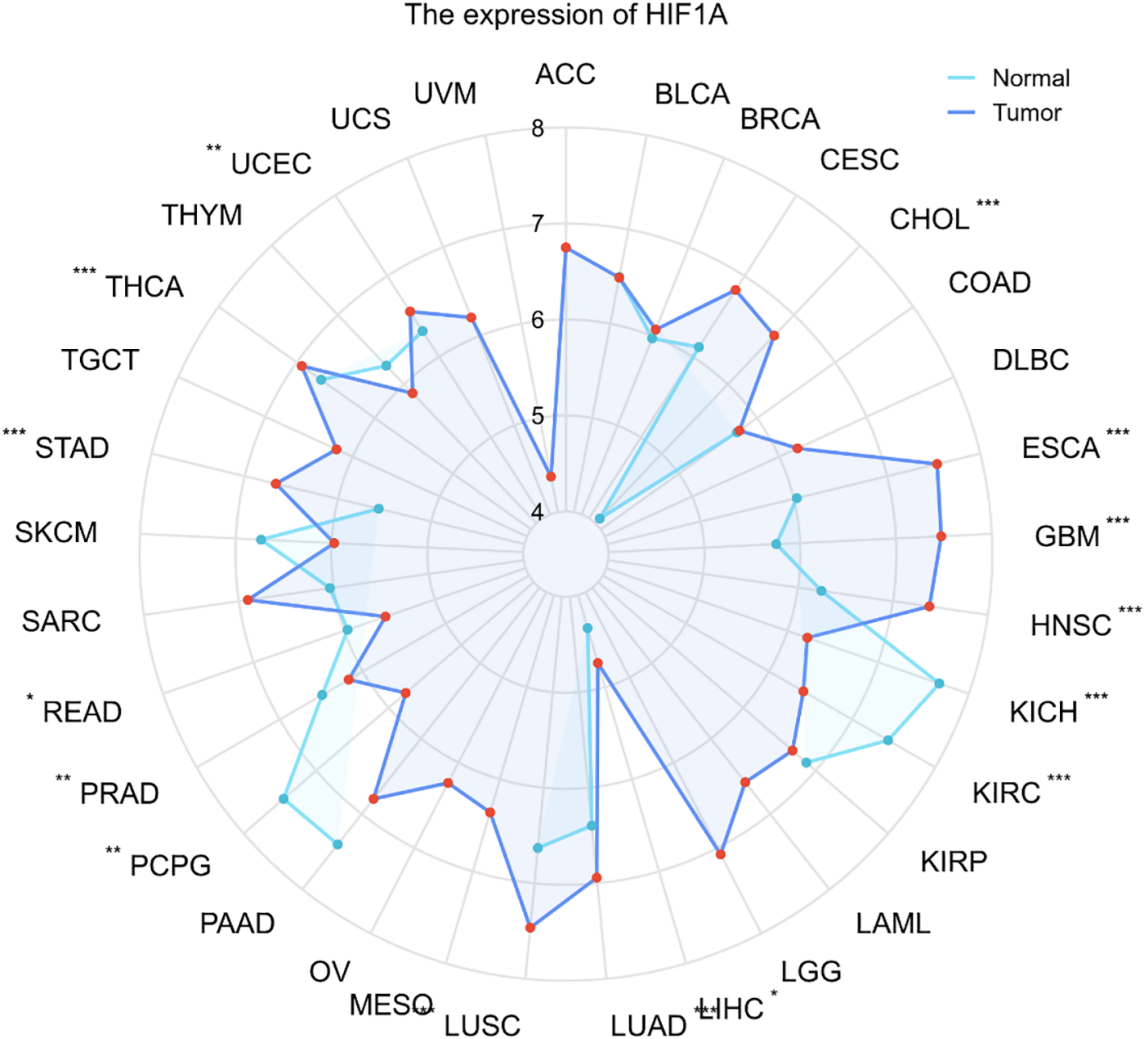
Pan-cancer expression profile of HIF1A across digestive system malignancies. This radar chart visualizes the relative expression levels of *HIF1A* mRNA in major gastrointestinal cancers based on TCGA pan-cancer analysis. The expression pattern highlights consistently elevated *HIF1A* in esophageal carcinoma (ESCA), gastric adenocarcinoma (STAD), hepatocellular carcinoma (HCC), and colorectal cancer (CRC), underscoring its broad oncogenic relevance(Statistical values of the target molecule were derived from TCGA datasets, and radar charts were employed to depict the variation of these statistical measures across different disease conditions. The Mann–Whitney U test (Wilcoxon rank sum test) was applied for statistical comparisons. All analyses were conducted using R (version 4.2.1) with the ggplot2 package (version 3.4.4) for visualization, Significance levels:* p < 0.05, ** p < 0.01, and *** p < 0.001).

HIF-1α is a master regulator of the cellular response to hypoxia, and its activation in the tumor microenvironment drives a core program of oncogenic processes. These canonical functions include metabolic reprogramming towards aerobic glycolysis (the Warburg effect) via upregulation of glucose transporters (e.g., GLUT1) and glycolytic enzymes (e.g., HK2, LDHA); promotion of angiogenesis through the induction of vascular endothelial growth factor (VEGF); and facilitation of invasion and metastasis by driving epithelial-mesenchymal transition (EMT). While these core mechanisms are common to most solid tumors, emerging evidence highlights that HIF-1α also engages in tumor type-specific, non-canonical pathways that contribute to unique malignant phenotypes and therapy resistance. Building upon pan-cancer analyses that reveal a consistent upregulation of HIF-1α across multiple malignancies, this section focuses on its central role in digestive system tumors, emphasizing these context-dependent mechanisms (**Figure 2**). The expression and activity of HIF 1α are markedly elevated in cancers such as esophageal, gastric, pancreatic, and hepatocellular, where it operates as a master regulator of tumor progression and therapy resistance. However, colon adenocarcinoma did not show significant findings in the figure. Nevertheless, after conducting a thorough literature review and synthesis, we identified existing studies on this topic. Therefore, we have supplemented and incorporated it into this section to enhance the completeness of the study. The following discussion outlines the tumor type specific pathways and networks governed by HIF-1α, providing a mechanistic basis for its potential as a therapeutic target in gastrointestinal oncology.

### 3.1 Esophageal cancer

In esophageal cancer, HIF-1α acts as a pivotal regulatory molecule that drives malignant progression through various mechanisms. Studies have shown that HIF-1α can form a complex with activating transcription factor 5 (ATF5), which together bind to the promoter regions of HIF-1 target genes, enhancing their transcriptional activity and thus promoting proliferation, migration, invasion, and EMT in esophageal cancer cells. Furthermore, HIF-1α directly upregulates the expression of CXCR4 by binding to the HRE in its promoter, thereby activating the CXCL12/CXCR4 chemotaxis axis and enhancing cell migration, invasion, and directional metastasis (45).

**Figure 3:**
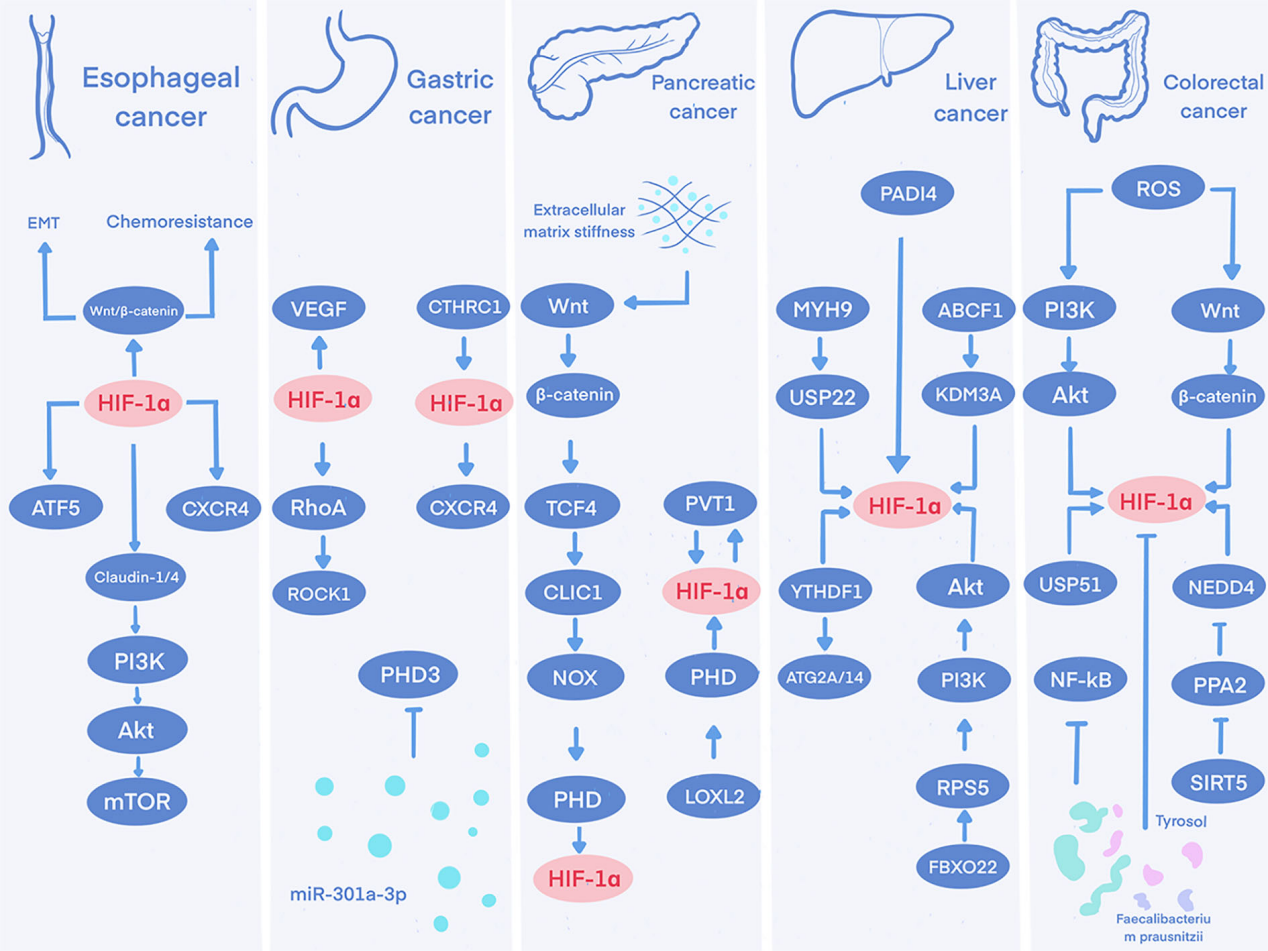
The multifaceted oncogenic network of HIF-1α in digestive system tumors. A mechanistic overview depicting how HIF-1α activation under hypoxia promotes tumor progression through diverse functions—including metabolic switch, angiogenesis, immune evasion, and drug resistance—across esophageal, gastric, pancreatic, hepatocellular, and colorectal carcinomas.

Conversely, HIF-1α establishes a positive feedback loop with the Wnt/β-catenin signaling pathway; it not only directly binds to the promoters of transcription factor 4 (TCF4)/transcription factor 7-like 2 (TCF7L2) to promote their expression but also physically interacts with and stabilizes β-catenin, cooperatively activating downstream target genes that further facilitate cell-cycle progression, EMT, and resistance to 5-fluorouracil (46). Simultaneously, in the hypoxic tumor microenvironment, HIF-1α modulates the expression of Claudin-1 and Claudin-4, influences the PI3K-Akt-mTOR pathway, and plays a role in regulating cell proliferation and apoptosis (47). Notably, in esophageal squamous cell carcinoma with lysine-specific demethylase 6A (KDM6A) deficiency, enhancer of zeste homolog 2 (EZH2) inhibitors can enhance radiosensitivity through HIF-1α-mediated epigenetic silencing of acyl-CoA synthetase long-chain family member 4 (ACSL4), thereby regulating ferroptosis (48). **Figure 3**.

### 3.2 Gastric cancer

In gastric cancer, hypoxia-inducible factor 1α (HIF-1α) occupies the center of a complex and precisely regulated network, with its activity and function controlled at multiple levels—from protein stability to transcriptional output.

First, at the protein stability level, the degradation of HIF-1α is directly modulated by upstream signaling. Studies show that extracellular matrix receptors can bind directly to HIF-1α and inhibit its ubiquitination, thereby blocking proteasomal degradation and effectively stabilizing HIF-1α protein levels under both normoxic and hypoxic conditions.

Second, at the functional output level, stabilized HIF-1α drives malignant phenotypes by activating diverse downstream pathways. On one hand, as a key transcription factor, it directly upregulates the expression of genes such as VEGF, promoting tumor angiogenesis. On the other hand, it activates the RhoA/ROCK1 signaling axis, triggering actin cytoskeleton reorganization and EMT, thereby enhancing tumor cell migration and invasion (13, 49). Additionally, collagen triple helix repeat containing 1 (CTHRC1) protein upregulates HIF-1α, which in turn increases the expression of key receptors such as CXCR4, further strengthening tumor cell motility and metastatic potential (50).

Moreover, the regulatory network becomes even more intricate. The hypoxic tumor microenvironment induces the expression of long non-coding RNA HIF1α-AS3, which competitively sponges miR-142-3p and miR-24-3p, leading to upregulation of prospero homeobox protein 1 (PROX1) gene expression and subsequent activation of key signaling pathways such as β-catenin. This mechanism promotes the reprogramming of mesenchymal cells into cancer-associated fibroblasts within the tumor stroma, thereby exacerbating chemotherapy resistance (51). In parallel, gastric cancer cells secrete exosomes enriched with miR-301a-3p under hypoxia. These exosomes inhibit PHD3-mediated hydroxylation and subsequent ubiquitin-dependent degradation of HIF-1α. This synergistic positive feedback loop between HIF-1α and miR-301a-3p promotes proliferation, invasion, migration, and EMT in gastric cancer (52).

Beyond its roles within cancer cells and cancer-associated fibroblasts(CAFs), HIF-1α profoundly influences the immune landscape of gastric cancer. It plays a critical role in the crosstalk between tumor cells and tumor-associated macrophages (TAMs). For instance, a lactate-driven mechanism involving MCT-HIF1α signaling promotes macrophage polarization toward an immunosuppressive M2-like phenotype (53). Furthermore, TAMs contribute to chemotherapy resistance in a HIF-1α-dependent manner. Treatment of gastric cancer cells with 5-fluorouracil (5-FU) induces the accumulation of reactive oxygen species and upregulates HMGB1 expression, which activates HIF-1α signaling. This process recruits macrophages that produce growth differentiation factor 15 (GDF15) into the tumor microenvironment, thereby exacerbating chemoresistance (54). Similarly, under cisplatin treatment, gastric cancer cells secrete leukemia inhibitory factor (LIF), activating the STAT3 pathway in macrophages and promoting their M2 polarization, which further contributes to chemotherapy resistance (55).

### 3.3 Pancreatic cancer

In the highly fibrotic and hypoxic microenvironment of pancreatic ductal adenocarcinoma (PDAC), the stabilization and activation of HIF-1α serve as the foundation for its core biological functions. The increased stiffness of the extracellular matrix (ECM) activates the Wnt/β-catenin signaling pathway, resulting in the nuclear translocation of the transcription factor TCF4, which directly upregulates the expression of chloride intracellular channel protein 1 (CLIC1). Through its ion-channel function, CLIC1 enhances the activity of NADPH oxidase (NOX), leading to elevated intracellular levels of ROS. The accumulated ROS inhibit prolyl hydroxylase (PHD) activity, resulting in impaired hydroxylation and subsequent ubiquitin-dependent degradation of HIF-1α, thereby significantly enhancing its protein stability (56). Concurrently, the transcriptional activity of HIF-1α is precisely regulated through a key positive feedback loop involving the long non-coding RNA PVT1. HIF-1α directly binds to the PVT1 promoter to drive its transcription, while PVT1, in turn, acts as a transcriptional co-activator, promoting HIF-1α expression and directly binding to the HIF-1α protein to protect it from proteasomal degradation (57).

However, the biological function of HIF-1α in PDAC exhibits significant context-dependency. Notably, in PDAC models with specific genetic backgrounds, HIF-1α displays tumor-suppressive properties: its loss upregulates protein phosphatase 1 regulatory subunit 1B (PPP1R1B), promotes phosphorylation of MDM2 at Ser166, enhances p53 ubiquitination and degradation, and consequently increases tumor invasiveness and metastatic potential, revealing the functional complexity of HIF-1α in PDAC (58).

When successfully activated, HIF-1α acts as a central hub driving malignant progression and therapy resistance in PDAC. One of its most prominent functions is inducing profound metabolic reprogramming, i.e., the Warburg effect. Lysyl oxidase-like 2 (LOXL2) stabilizes HIF-1α by generating hydrogen peroxide that interferes with PHD-dependent hydroxylation, thereby promoting the transcription of multiple glycolytic genes. This enables tumor cells to preferentially utilize glycolysis—even under aerobic conditions—to meet the energetic and biosynthetic demands of rapid proliferation (59).

In chemotherapy resistance, the central role of HIF-1α is particularly evident. Recent studies show that HIF-1α directly upregulates the transcription of *AVL9*. The AVL9 protein acts as a scaffold to promote the binding of IκBα to SKP1, exacerbating IκBα ubiquitination and degradation. This leads to aberrant activation of the NF-κB signaling pathway and ultimately mediates resistance to the gemcitabine plus albumin-bound paclitaxel (AG) regimen (60). This mechanism coexists with other known pathways; for example, loss of the tumor suppressor methylthioadenosine phosphorylase (MTAP) activates serine/threonine kinase RIO kinase 1 (RIOK1), which phosphorylates and stabilizes HIF-1α, promoting *de novo* purine synthesis and glycolysis, and consequently inducing gemcitabine resistance (61). In contrast, negative feedback mechanisms also exist to inhibit HIF-1α. The anti-inflammatory cytokine IL-37, upon binding to its receptor IL-18Rα/IL-1R8, inhibits STAT3 phosphorylation and downregulates HIF-1α expression, whereas HIF-1α can reciprocally suppress IL-37 transcription (62).

These seemingly paradoxical findings collectively depict the dual role of HIF-1α in PDAC: acting as a tumor suppressor in specific genetic contexts via the PPP1R1B-p53 axis to inhibit metastasis, while promoting tumor progression and chemotherapy resistance in other settings through metabolic reprogramming, NF-κB activation, and related pathways.

### 3.4 Hepatocellular carcinoma

HIF-1α occupies a central regulatory position in the progression of hepatocellular carcinoma, with its stability and activity governed by a complex network of post-translational modifications and protein-protein interactions. Research indicates that under microenvironmental stresses, such as hypoxia or nutrient deprivation, HIF-1α can evade conventional ubiquitin-mediated degradation through various mechanisms. For instance, phosphorylated myosin heavy chain 9 (MYH9)(p-MYH9, Ser1943) recruits the deubiquitinase ubiquitin-specific peptidase 22 (USP22), which stabilizes HIF-1α and promotes cancer stemness features and lenvatinib resistance (63). Concurrently, modifications of HIF-1α itself are critical; peptidyl arginine deiminase 4 (PADI4)-mediated citrullination at the R698 site of HIF-1α impedes its binding to VHL, thereby inhibiting its ubiquitination and degradation (64). Furthermore, lactylation at the K430 site of ATP-binding cassette subfamily F member 1 (ABCF1) enhances its nuclear translocation, where it upregulates lysine-specific demethylase 3A (KDM3A) and activates the HIF-1α transcriptional signaling axis, thereby establishing a metabolic signal amplification loop (65).

Furthermore, HIF-1α acts as an upstream transcription factor to regulate the expression of downstream effectors. For instance, it directly induces the transcription of the m6A reader YTHDF1, which subsequently enhances the translation of autophagy related 2A (ATG2A) and autophagy related 14 (ATG14), promoting autophagy and thereby supporting tumor survival and progression (66). In parallel, the E3 ubiquitin ligase F-box protein 22 (FBXO22) ubiquitinates and degrades ribosomal protein S5 (RPS5), relieving its inhibition of the PI3K/AKT pathway. This activation further promotes the HIF-1α/VEGF-A axis, facilitating angiogenesis and metastasis (67).

### 3.5 Colorectal cancer

In colorectal cancer (CRC), the aberrant upregulation of HIF-1α is driven by multiple interconnected mechanisms that extend beyond classic hypoxia. The accumulation of reactive oxygen species (ROS) within the tumor microenvironment activates the PI3K/Akt pathway and aberrantly stimulates Wnt/β-catenin signaling, leading to both the stabilization of the HIF-1α protein and the transcriptional enhancement of its expression (68). This intricate regulation is further fine-tuned at the post-translational level; the deubiquitinase ubiquitin-specific peptidase 51 (USP51) directly binds to and deubiquitinates HIF-1α, stabilizing it and establishing a positive feedback loop that amplifies its activity. Conversely, the E3 ubiquitin ligase neural precursor cell expressed, developmentally down-regulated 4 (NEDD4) mediates HIF-1α degradation—a process facilitated by inorganic pyrophosphatase 2 (PPA2)—though this can be counteracted under hypoxia by sirtuin 5 (SIRT5)-mediated desuccinylation of PPA2, which promotes HIF-1α stabilization and metastatic potential (69, 70).

Functionally, HIF-1α acts as a central driver of the Warburg effect in CRC. It transcriptionally upregulates key glycolytic genes such as GLUT1, HK2, and LDHA, shifting energy metabolism toward glycolysis and the pentose phosphate pathway. This metabolic reprogramming not only fuels rapid tumor growth but also directly contributes to chemoresistance against 5-fluorouracil (5-FU). Furthermore, HIF-1α engages in a positive feedback loop with the mTORC1 pathway (HIF-1α → RRAGB → mTORC1 → HIF-1α), which cooperatively promotes tumor progression and survival (71).

This pro-tumorigenic role of HIF-1α is further amplified through intricate crosstalk with the tumor microenvironment, where it orchestrates immunosuppressive and therapy-resistant niches. For instance, HIF-1α is central to a bidirectional interaction with tumor-associated macrophages (TAMs). On one hand, CD206^+^ M2-polarized TAMs secrete TGF-β, which enhances glycolysis in colon cancer cells, leading to HIF-1α upregulation and subsequent activation of the TRIB3/β-catenin/Wnt axis, thereby bolstering cancer cell invasiveness (72). On the other hand, the hypoxic and glycolytic tumor milieu results in lactate accumulation. Macrophages within this environment upregulate monocarboxylate transporter 1 (MCT1), facilitating lactate uptake, which stabilizes HIF-1α and initiates the HIF-1α/STAT3/SPP1 pathway, driving their own polarization toward a pro-tumor M2-like phenotype. This reciprocal activation creates a vicious cycle that sustains malignant progression (16). Moreover, under chemotherapeutic stress like 5-FU treatment, CRC cells release HMGB1 in a ROS-dependent manner, activating HIF-1α signaling that recruits GDF15-producing TAMs to further exacerbate chemoresistance (73).

HIF-1α also serves as a critical node in the communication between cancer cells and cancer-associated fibroblasts (CAFs), a relationship that underpins therapy resistance. CAF-derived TGF-β1 upregulates the long non-coding RNA WARS2-IT1 in CRC cells. This lncRNA disrupts the PHD2-HIF-1α interaction, inhibiting HIF-1α hydroxylation and enhancing its stability. The resultant increase in HIF-1α activity drives glycolysis, conferring resistance to radiotherapy (74). In a parallel pathway, CAF-secreted TGF-β2 synergizes with HIF-1α to upregulate the Hedgehog transcription factor GLI family zinc finger 2 (GLI2) in cancer cells, thereby enhancing their tolerance to chemotherapeutic agents (75).

Adding another layer of complexity, HIF-1α within myeloid-derived suppressor cells (MDSCs) orchestrates a potent immunosuppressive network. It promotes their immunosuppressive function by upregulating Nos2 and Arg1 while downregulating ROS production, and also steers their differentiation toward a TAM-like phenotype (76). Most notably, HIF-1α directly binds to the hypoxia-response element in the promoter of the immune checkpoint VISTA, driving its specific expression on MDSCs within hypoxic tumor regions. This HIF-1α-VISTA axis is a key mediator of MDSC-induced T-cell suppression and an independent predictor of poor overall survival in CRC patients, representing a critical mechanism of immune evasion (15).

Finally, the gut microbiota introduces an additional layer of regulation by modulating HIF-1α activity. Levels of the beneficial bacterium *Faecalibacterium prausnitzii* and its associated metabolite tyrosol are markedly reduced in CRC patients. Functionally, tyrosol suppresses CRC cell growth by simultaneously inhibiting the NF-κB and HIF-1α signaling pathways: it reduces phosphorylated p65 and IκB levels while downregulating HIF-1α and HIF-1β expression, thereby alleviating inflammation and oxidative stress within the tumor microenvironment (77). As a major component of extra virgin olive oil, dietary tyrosol supplementation, in synergy with *F. prausnitzii*, exerts anti-tumor effects (78). These findings reveal a novel axis by which host-microbiota interactions can restrain CRC progression through direct targeting of the HIF-1α/NF-κB pathway.

## 4 Therapeutic targeting of HIF-1α and associated pathways

Given the central role of HIF−1α in orchestrating diverse pathological processes across digestive system inflammation and malignancies, targeting its regulatory network represents a promising therapeutic avenue. The multifaceted functions of HIF−1α—ranging from metabolic reprogramming and angiogenesis to immune modulation and therapy resistance—offer numerous nodes for pharmacological intervention. Strategies currently under investigation include direct inhibition of HIF−1α itself (e.g., with echinomycin, PX−478, or acriflavine), modulation of its stability (e.g., PHD inhibitors or activators), and blockade of its downstream effectors (e.g., VEGF, GLUT1, LDHA, NF−κB, STAT3, or specific long non−coding RNAs). However, the context−dependent and cell−type−specific roles of HIF−1α (e.g., protective in intestinal epithelium versus pro−tumorigenic in cancer cells) necessitate careful consideration of the therapeutic window and combination strategies. (**Table 1**) compiles the key pathways described in this review and highlights corresponding therapeutic targets that could be exploited to intervene in digestive system diseases.

**Table 1:** Key HIF-1α-associated pathways and potential therapeutic strategies in digestive system pathologies.

Disease	Cell type / Mechanism	Pathway / Molecular axis	Potential therapeutic strategy
Periodontitis	Periodontal ligament cells	TNF-α/IL-1β → UCHL1 → YAP → VEGFA/angiopoietin-1	UCHL1 inhibitors; HIF-1α inhibitors; anti-VEGF antibodies (21, 22)
		LINC01126 / miR-518a-5p / HIF-1α / MAPK (p38, JNK, ERK) → inflammation, apoptosis	LINC01126 silencing; miR-518a-5p mimics; MAPK inhibitors (23)
Pulpitis	Dental pulp stem cells	miR-143-5p / HIF-1α / RORA → angiogenesis	miR-143-5p mimics; HIF-1α inhibitors (24)
		miR-22-3p targets NLRP3 and HIF-1α → NLRP3/CASP1 inflammasome inhibition	miR-22-3p mimics; NLRP3 inhibitors (e.g., MCC950) (25)
	Gingival fibroblasts	Hypoxia + *P. gingivalis* LPS → NLRP3 inflammasome activation	NLRP3 inhibitors; anti-inflammatory agents (26)
Eosinophilic esophagitis	Esophageal epithelial cells	HIF-1α dysregulation → ↓CLDN1, GLUT1, PGK1, ADM, VEGFA, ENO1 → barrier dysfunction	HIF-1α stabilizers (e.g., PHD inhibitors) to restore protective function (27)
		CD73 / adenosine / ADORA2B / cAMP / CREB → tight junction impairment	CD73 agonists; ADORA2B agonists (28)
		HIF-1α knockdown → OXPHOS ↑, glycolysis ↓ → differentiation defect	Metabolic modulators; HIF-1α activators (29)
Hepatitis B	Hepatocytes	HIF-1α stabilization → RelB ↓ → A3B ↓ → HBV persistence	HIF-1α inhibitors (32); strategies to restore RelB/A3B axis (32, 33)
Chronic pancreatitis	Pancreatic acinar cells	HIF-1α → SPHK1 / S1P / S1PR2 → AMPK-mTOR → PSC autophagy and fibrosis	SPHK1 inhibitors (e.g., SKI-II); S1PR2 antagonists; mTOR inhibitors (e.g., rapamycin) (35)
Autoimmune pancreatitis	Macrophages / PSCs	HIF1A+ macrophages → NAMPT/visfatin → TLR4 / ROS / TGF-β / Smad3 → PSC activation	NAMPT inhibitors (e.g., FK866); TLR4 antagonists; TGF-β inhibitors (e.g., galunisertib) (36)
Crohn’s disease	T cells / epithelial cells	HIF-1α / NF-κB → VEGF, ABC transporters (MDR1, MRP4) → Th17 dysfunction	HIF-1α inhibitors; NF-κB inhibitors (e.g., BAY 11-7082); ABC transporter modulators (38–40)
Ulcerative colitis	Intestinal epithelial cells	HIF-1α binds GPX4 promoter → inhibits ferroptosis	HIF-1α activators (protective, but caution in long-term use) (7, 41)
	CD4+ T cells	mTORC1 / HIF-1α / Glut1 / RORγt / Foxp3 → Th17/Treg imbalance	mTOR inhibitors (e.g., everolimus); HIF-1α inhibitors; Glut1 inhibitors (42)
	Neutrophils	CsA → SIRT6 / HIF-1α / PFKFB3 / PDK4 → metabolic reprogramming	SIRT6 modulators; PFKFB3 inhibitors (e.g., PFK15) (44)
Esophageal cancer	Cancer cells	HIF-1α / ATF5 complex → proliferation, migration, EMT	ATF5 inhibitors; HIF-1α inhibitors (45)
		HIF-1α binds CXCR4 HRE → CXCL12/CXCR4 axis	CXCR4 antagonists (e.g., AMD3100) (45)
		HIF-1α / Wnt/β-catenin loop (TCF4, β-catenin) → 5-FU resistance	Wnt inhibitors (e.g., LGK974); β-catenin inhibitors; combination with 5-FU (46)
		HIF-1α regulates Claudin-1/4 via PI3K-Akt-mTOR	PI3K/Akt/mTOR inhibitors (e.g., BEZ235) (47)
	Cancer cells (KDM6A-deficient)	EZH2 inhibitor → HIF-1α-mediated ACSL4 silencing → ferroptosis	EZH2 inhibitors (e.g., tazemetostat); ferroptosis inducers (48)
Gastric cancer	Cancer cells	Extracellular matrix receptor inhibits HIF-1α ubiquitination	Targeting matrix receptors (e.g., integrin antagonists) (49)
		HIF-1α → VEGF	VEGF inhibitors (e.g., bevacizumab) (13, 49)
		HIF-1α → RhoA/ROCK1 → EMT	ROCK inhibitors (e.g., Y-27632) (13, 49)
		CTHRC1 upregulates HIF-1α → CXCR4	CTHRC1 targeting; CXCR4 antagonists (50)
	CAFs	LncRNA HIF1A-AS3 sponges miR-142-3p/miR-24-3p → PROX1 → β-catenin → CAF reprogramming	HIF1A-AS3 silencing; miRNA mimics; β-catenin inhibitors (51)
	Cancer cells / exosomes	Hypoxic exosomes miR-301a-3p inhibit PHD3 → HIF-1α stabilization	miR-301a-3p inhibitors; PHD3 activators (52)
	Macrophages	Lactate / MCT-HIF1α → M2 TAM polarization	MCT1/4 inhibitors (e.g., AZD3965); HIF-1α inhibitors (53)
	TAMs	5-FU → ROS/HMGB1/HIF-1α → recruit GDF15-producing TAMs → chemoresistance	HMGB1 inhibitors (e.g., glycyrrhizin); GDF15 blocking antibodies (54)
		Cisplatin → LIF / STAT3 → M2 TAM	LIF inhibitors; STAT3 inhibitors (e.g., napabucasin) (55)
Pancreatic cancer	Cancer cells	ECM stiffness → Wnt/β-catenin/TCF4 → CLIC1 → NOX/ROS → PHD inhibition → HIF-1α stabilization	CLIC1 inhibitors; NOX inhibitors (e.g., GKT137831); Wnt inhibitors (56)
		LncRNA PVT1 / HIF-1α positive feedback	PVT1 silencing; HIF-1α inhibitors (57)
		HIF-1α loss → PPP1R1B ↑, p-MDM2 (Ser166) → p53 degradation → metastasis	MDM2 inhibitors (e.g., nutlin-3a);p53 reactivators (58)
		LOXL2 stabilizes HIF-1α → glycolysis genes	LOXL2 inhibitors (e.g., PXS-5153A)[59]
		HIF-1α upregulates AVL9 → IκBα degradation → NF-κB activation → AG chemotherapy resistance	AVL9 inhibitors; NF-κB inhibitors (e.g., bortezomib) (60)
		MTAP loss → RIOK1 stabilizes HIF-1α → purine synthesis/glycolysis → gemcitabine resistance	RIOK1 inhibitors; combination with antimetabolites (61)
		IL-37 / STAT3 / HIF-1α negative feedback	IL-37 agonists (e.g., recombinant IL-37) (62)
Hepatocellular carcinoma	Cancer cells	p-MYH9 recruits USP22 → deubiquitinates HIF-1α → stemness, lenvatinib resistance	USP22 inhibitors; MYH9 phosphorylation inhibitors (63)
	Cancer cells	PADI4 citrullinates HIF-1α (R698) → inhibits VHL binding → HIF-1α stabilization	PADI4 inhibitors (e.g., GSK484) (64)
		ABCF1 lactylation (K430) → nuclear translocation → KDM3A → HIF-1α activation	Targeting lactylation; KDM3A inhibitors (65)
		HIF-1α induces YTHDF1 → ATG2A/ATG14 translation → autophagy	YTHDF1 inhibitors (66)
		FBXO22 degrades RPS5 → PI3K/AKT → HIF-1α/VEGF-A → angiogenesis	FBXO22 inhibitors; PI3K/AKT inhibitors; VEGF inhibitors (67)
Colorectal cancer	Cancer cells	ROS/PI3K/Akt and Wnt/β-catenin → HIF-1α upregulation	Antioxidants; PI3K inhibitors (e.g., alpelisib); Wnt inhibitors (68)
		USP51 deubiquitinates HIF-1α	USP51 inhibitors (69)
		SIRT5 desuccinylates PPA2 → inhibits NEDD4-mediated degradation	SIRT5 inhibitors (e.g., MC3482); enhance NEDD4 activity (70)
		HIF-1α → GLUT1, HK2, LDHA → Warburg effect, 5-FU resistance	GLUT1 inhibitors (e.g., WZB117); HK2 inhibitors (e.g., 3-BrPA); LDHA inhibitors (e.g., FX11) (66)
		HIF-1α / RRAGB / mTORC1 loop	RRAGB inhibitors; mTORC1 inhibitors (71)
	TAMs (M2)	TGF-β → glycolysis/HIF-1α/TRIB3/β-catenin	TGF-β inhibitors (e.g., galunisertib); TRIB3 targeting (72)
		Lactate / MCT1 / HIF-1α / STAT3 / SPP1 → M2 TAM	MCT1 inhibitors; STAT3 inhibitors (16)
		5-FU → ROS/HMGB1/HIF-1α → recruit GDF15-producing TAMs	HMGB1 inhibitors; GDF15 blocking antibodies (73)
	CAFs	TGF-β1 → lncRNA WARS2-IT1 → inhibits PHD2 → HIF-1α stabilization → radioresistance	TGF-β inhibitors; WARS2-IT1 silencing; PHD2 activators (74)
		TGF-β2 + HIF-1α → GLI2 → chemoresistance	GLI2 inhibitors (e.g., GANT61) (75)
	MDSCs	HIF-1α → Nos2, Arg1, ↓ROS → immunosuppression	HIF-1α inhibitors; MDSC-targeting agents (76)
		HIF-1α binds VISTA promoter → VISTA expression → T cell suppression	VISTA antibodies (e.g., CA-170); HIF-1α inhibitors (15)
	Gut microbiota	*Faecalibacterium prausnitzii* / tyrosol → inhibits NF-κB and HIF-1α	Probiotics; tyrosol supplementation (77, 78)

## 5 Conclusion

This review systematically highlights the central role of HIF-1α as a critical hub in digestive system pathology. Beyond serving as a key mediator of hypoxia response, it plays an important role in linking inflammation and tumorigenesis. In inflammatory conditions, sustained HIF-1α activation disrupts tissue homeostasis by modulating inflammatory mediators and reprogramming cellular metabolism, thereby exacerbating or perpetuating inflammatory states. In malignancies, HIF-1α not only directly promotes malignant phenotypes of cancer cells but also remodels the tumor microenvironment—such as by polarizing tumor-associated macrophages and activating fibroblasts—to foster an immunosuppressive milieu, which facilitates tumor progression and influences responses to immunotherapy. Targeting the HIF-1α pathway thus represents a promising strategy for intervening in these diseases, for example by inhibiting its activity to reverse immunosuppression and enhance the efficacy of existing therapies. However, the cell-type-specific functions of HIF-1α and its complex downstream networks remain major challenges for precise intervention. Future studies should focus on elucidating its context-dependent roles and developing targeted combination therapies, with the goal of achieving breakthroughs in the management of digestive inflammatory diseases and cancers.
